# Visual Dysfunction in Parkinson’s Disease

**DOI:** 10.3390/brainsci13081173

**Published:** 2023-08-07

**Authors:** Francisco Nieto-Escamez, Esteban Obrero-Gaitán, Irene Cortés-Pérez

**Affiliations:** 1 Department of Psychology, University of Almeria, 04120 Almeria, Spain; 2 Center for Neuropsychological Assessment and Rehabilitation (CERNEP), 04120 Almeria, Spain; 3 Department of Health Sciences, University of Jaen, Paraje Las Lagunillas s/n, 23071 Jaen, Spain; icortes@ujaen.es

**Keywords:** Parkinson’s disease, visual impairment, visuoperceptive deficit, visuospatial deficit, visual hallucinations, dopamine

## Abstract

Non-motor symptoms in Parkinson’s disease (PD) include ocular, visuoperceptive, and visuospatial impairments, which can occur as a result of the underlying neurodegenerative process. Ocular impairments can affect various aspects of vision and eye movement. Thus, patients can show dry eyes, blepharospasm, reduced blink rate, saccadic eye movement abnormalities, smooth pursuit deficits, and impaired voluntary and reflexive eye movements. Furthermore, visuoperceptive impairments affect the ability to perceive and recognize visual stimuli accurately, including impaired contrast sensitivity and reduced visual acuity, color discrimination, and object recognition. Visuospatial impairments are also remarkable, including difficulties perceiving and interpreting spatial relationships between objects and difficulties judging distances or navigating through the environment. Moreover, PD patients can present visuospatial attention problems, with difficulties attending to visual stimuli in a spatially organized manner. Moreover, PD patients also show perceptual disturbances affecting their ability to interpret and determine meaning from visual stimuli. And, for instance, visual hallucinations are common in PD patients. Nevertheless, the neurobiological bases of visual-related disorders in PD are complex and not fully understood. This review intends to provide a comprehensive description of visual disturbances in PD, from sensory to perceptual alterations, addressing their neuroanatomical, functional, and neurochemical correlates. Structural changes, particularly in posterior cortical regions, are described, as well as functional alterations, both in cortical and subcortical regions, which are shown in relation to specific neuropsychological results. Similarly, although the involvement of different neurotransmitter systems is controversial, data about neurochemical alterations related to visual impairments are presented, especially dopaminergic, cholinergic, and serotoninergic systems.

## 1. Introduction

Parkinson’s disease (PD) is the second most common neurodegenerative disorder after Alzheimer’s disease, with an estimated prevalence for industrialized countries of 1% of the population over 60 years and 3% in people older than 80 years [1]. PD is characterized by the loss of dopaminergic neurons in substantia nigra pars compacta, which modulate the fronto–thalamo–striatal circuit, leading to a wide range of motor and non-motor symptoms (NMSs) [2,3]. Thus, NMS is a broad spectrum of symptoms, including mood disorders [4], sensory and perceptual dysfunction [5], and cognitive disturbances [6], with visuospatial processing impairments among the NMSs having accumulated the most interest in the last years [7,8].

Basic visual processes are affected in PD, including reduced spatial contrast sensitivity and impaired color discrimination [9], oculomotor control defects and diplopia [10,11], dry eyes disease [12], glaucoma [13], and visual hallucinations even in the absence of dementia [14]. Furthermore, visual pathologies are accompanied by a poor performance in tasks requiring high-order processing capabilities, such as object mental rotation [15], perception of space [16], spatial maps representation, visuospatial working memory, effective navigation, and target localization [17,18,19], which can be considered as preclinical markers and predictors of disease development [20].

Impaired visual and visuospatial functions can affect a broad range of essential daily living skills, such as driving, reading, writing, or walking [21,22]. These problems have been reported to be increased throughout the progress of the illness, resulting in a reduction of self-efficacy and quality of life.

This review aims to provide a comprehensive review of ocular, visuoperceptive, and visuospatial disturbances in PD, as well as the consequences of integration deficits between high-level perceptual and cognitive processes. For that purpose, published research employing specific neuropsychological tasks have been included, and the disease’s effects on their performance have been examined, even in different phases of the disease. The neurobiological substrates, including structural and functional alterations, and neurochemical mechanisms will be revised for each condition. Until now, some papers reviewing visual disturbances in PD have been published. Some time ago, an outstanding paper by Weil et al. [20] revised changes in visual function at different stages of visual processing, paying special attention to genetic factors, and the relation between clinical features of PD and visuoperceptual alterations as a biomarker of the disease. And, more recently, another remarkable review by Ghazi-Saidi [23] has analyzed the relation between visuospatial and executive deficits in PD. Our work is an attempt to provide an actual and comprehensive picture of visual dysfunctions in PD. 

## 2. Ocular and Visual Impairments in Parkinson’s Disease

PD patients show a number of ocular and visual impairments resulting from pathological processes or consequences of medication, and usually get worse during disease progression [24], with up to 70% of patients reporting recurrent visual complaints [25]. Thus, a panoply of oculomotor issues have been described in PD patients [10,26], including poor binocular convergence, double vision (diplopia), bradykinesia and hypokinesia of ocular pursuit, impaired vertical upward and downward glaze, defective saccadic movements with longer reaction times, hypometria, and square wave jerks [24,27,28,29]. These problems have direct consequences on patients’ abilities to perform daily tasks, such as reading. writing, driving, or navigating [5]. Furthermore, visual deficits can even be found in the prodromal stages of PD, as early as decades before the onset of motor symptoms [30].

### 2.1. Retinal Changes

**Figure 1:**
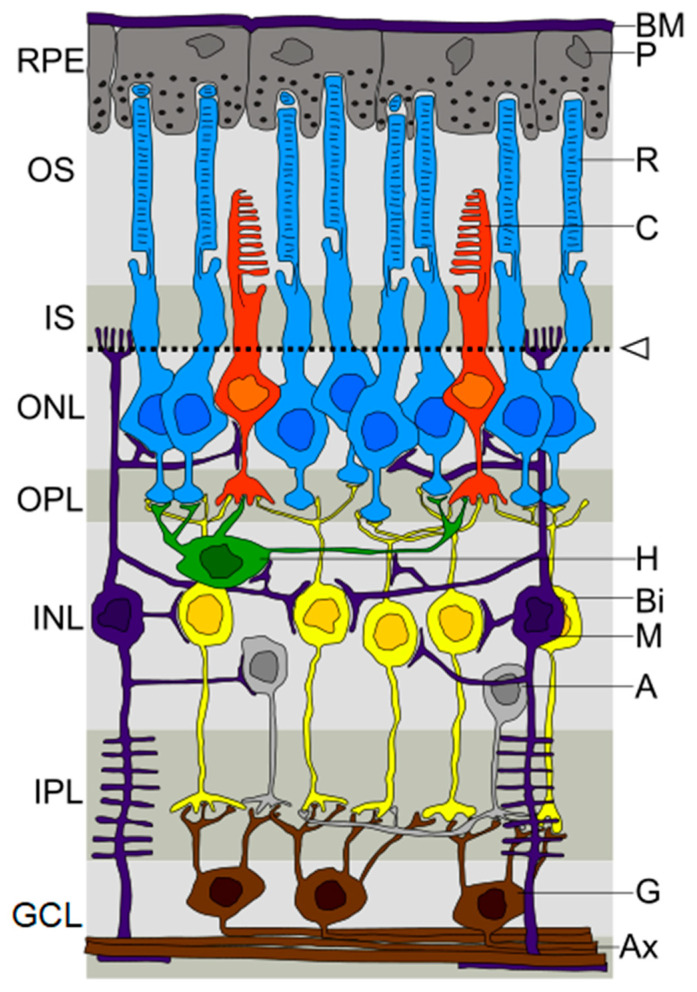
Portion of a human retina. A: Amacrine cell; Bi: Bipolar cell; BM: Bruch’s membrane; C: Cone; GCL: Ganglion cell layer; H: Horizontal cell; INL: Inner nuclear layer; IPL: Inner plexiform layer; IS: Inner segment; M: Muller cell; ONL: Outer nuclear layer; OPL: Outer plexiform layer; OS: Outer Segment; P: Pigment epithelial cell; R: Rod; RPE: Retinal pigment epithelium; G: Ganglion cell; AX: Axons. Adapted from Hartmann and Schmid [43]. Image licensed under GFDL by the author. https://de.wikipedia.org/wiki/Datei:Retina.jpg (accessed on 19 June 2023).

In the PD population, the main structures that make up the eyeball and the optic nerve are quantitatively and qualitatively affected. High-resolution structural imaging approaches by optical coherence tomography (OCT) of the retina show a decrease of the macular retinal thickness, macular volume, average retinal nerve fiber layer (RNFL), retinal ganglion cell layer (RGC), inner plexiform layer (IPL), inner nuclear layer (INL), outer plexiform layer (OPL), outer nuclear layer (ONL), retinal pigment epithelium, and photoreceptor layer in PD patients [31,32,33,34] (See Figure 1). However, macular thickness or volume are not always reduced [33,35].

Some authors have attempted to correlate retinal layer thinning with clinical scales scores. It has been reported that RNFL thickness reduction correlated to duration and severity of disease [35,36]. However, other authors could not confirm this alteration [37,38,39] nor its relation with disease duration or severity [40]. The RGCIPL layer thickness in the parafovea has been the parameter most frequently correlated with visual outcomes in PD patients [40,41,42].

Along with structural alterations, functional changes assessed through the visual evoked response (VER) and the electroretinogram (ERG) have also been shown to correlate with PD duration and severity [35]. It has also been proposed that electrophysiological alterations begin years before structural changes are observable [44].

Retinal degeneration may be caused by progressive dopamine depletion and α-synuclein-mediated axonal degeneration [13]. Dopamine is a key neurotransmitter in the retina, and its depletion results in a reduced electrical response under different light conditions [45]. IPL and GCL thinning observed in PD has been linked to dopaminergic loss in the left substantia and disease severity [46]. Furthermore, dopamine also influences other retinal neurotransmitters involved in retinal processing, such as glutamate, GABA, and glycine, disrupting these neurotransmission pathways [44].

Moreover, misfolded α-synuclein [47] and phosphorilated α-synuclein [48] have been reported in the inner retinal layer, and retinal α-synucleinopathy density scores positively correlate with brain α-synucleinopathy density scores, pathology stage, and the UPDRS-III motor sub-score [49].

Retinal and macula nerve fiber layer thinning is also linked with minor hallucinations [50] and disease duration and severity [51]. Moreover, retinal layer thinning correlates with frontal and occipital cortex thickness and is linked to lower scores in the Poppelreuter-Ghent overlapping figures test and the RPMT in a visually impaired PD sample [52]. Recently, Hannaway et al. [53] have reported that visual dysfunction is a better predictor than retinal thickness for dementia in PD. 

### 2.2. Glaucomatous Disturbances 

There is also a higher incidence of glaucoma and glaucomatous-like visual field defects, with the majority of cases related to open-angle glaucoma [54,55]. However, epidemiological data are scarce, and the evidence regarding these deficits is low [56,57].

### 2.3. Pupil Reactivity

Alterations in pupil reactivity have been observed in PD patients. PD patients show a significantly lager pupil diameter, with unequal pupil size after light adaptation and longer light reflex latencies and constriction times, along with reduced contraction amplitude [26,58]. Some studies suggest that pupil changes could be independent from dopaminergic deficiency [59], and dopaminergic treatment has no effect on the pupil light reflex [60]. Alhassan et al. [59] suggest that both parasympathetic (cholinergic) and sympathetic (adrenergic) autonomic systems are altered in PD, but the parasympathetic pathway is more affected. The parasympathetic imbalance is considered an early manifestation of PD [61]. You et al. [62] point out that pupillary parasympathetic dysfunction advances with the progression of PD, whereas pupillary sympathetic dysfunction changes slowly.

Pupil reactivity alterations may also reflect a sensory deficit due to impaired retinal or optic nerve function, but it has also been suggested they could be due to the degeneration of subcortical regions, such as the locus coeruleus in the brain stem [59]. 

Finally, cognitively impaired PD patients show more pupil constriction deficits than those with normal cognitive functions, similarly to pupil dysfunction reported in Alzheimer’s disease [63]. Kahya et al. [64] have reported an increased pupillary response with increased postural demand in PD.

### 2.4. Eyelid and Blink Reflex

Eyelid impairments, including a reduction of blink rate, apraxia in the opening of the eyelid, blepharospasm, and ptosis of the upper and Meibomian gland disease have been reported [26,65,66,67]. Three types of eyelid movement abnormalities are notable in the experimental models of PD: blink hyperreflexia, impaired blink reflex plasticity, and reduced rate and impaired rhythm of the spontaneous blinks [68].

Blepharospasm (BSP) is a form of focal dystonia that manifests with spasms of the eyelids, involuntary closure of the eye, and enhanced spontaneous blinking, or any combination of the previous ones [69]. 

Armstrong [70] reported that blink duration and excitability appear to be increased, which may be related to a loss of dopaminergic neurons. Furthermore, many PD patients show a reduced habituation response of the blink reflex, which may improve after treatment with levodopa or amantidine [70], although other authors reported no positive relationship between blink rate and dopamine synthesis capacity [71].

A reduced corneal sensitivity has been reported in PD by Reddy et al. [72]. And, some authors have shown that the lens can show defects in PD [12], with an increased prevalence of marked nuclear cataracts and a higher intensity of subscapular cataracts.

One of the most usual oculo-visual conditions is dry eye disease, affecting up to 60% of PD patients [12,67]. Dry eye disease in PD can be the result of corneal hypoesthesia, decreasing blinking rate and reflex lacrimation, autonomic neuropathy leading to decreased tear secretion, increased tear osmolarity, decreased tear mucin, and lipid layer disruption secondary to meibomian gland dysfunction [67]. 

### 2.5. Visual Acuity, Contrast Sensitivity, and Color Vision in PD

PD patients often complain of poor vision. This deficit can result from, besides other reasons, impaired visual acuity, with low-contrast acuity particularly affected [73]. A reduction of contrast sensitivity has been reported, particularly for intermediate and high frequencies [27] in central (foveal) and peripheral locations [74]. Loss of color vision has also been described [75]. Cross-sectional studies show that PD patients self-report poor eyesight [76] and have poorer objectively measured visual function parameters [77]. 

Polo et al. [77] found that parameters corresponding to visual acuity at different contrast levels and all contrast sensitivity test results were altered in patients with PD in comparison with healthy participants, with contrast sensitivity the most affected variable. These authors also reported that patients had a tendency to protanomaly.

Polo et al. [77] also proposed that RGC loss could be the cause of contrast and color deficiencies in PD, with dopaminergic D1 and D2 receptors playing a role in color vision and contrast sensitivity alterations [32]. And, reduced macular thickness and volume have been associated with poor visual acuity, contrast sensitivity, and color vision [77].

Although a retinal dopamine deficit might be related to contrast sensitivity deficits, the orientation-specific impairment points to cerebral cortex involvement [20]. Contrast sensitivity deficits can be partially reversed with levodopa, and apomorphine has been shown to improve contrast perception at all spatial frequencies [78]. 

Color discrimination deficits may be an early dopaminergic symptom in PD and a disease-specific feature [75]. Red–green color blindness (protan–deutan axis) produces blurred vision, with reduced perception of monochromatic contours, especially for dark green, light blue, and dark red stimuli [20]. Color vision dysfunction is observed even at early stages of the disease and progresses with the disease [79], affecting the motor speed and core region of the body performance [73]. 

Recently, dysfunction across all aspects of vision, including visual acuity, contrast sensitivity, color vision, and higher-order visual processes, have been linked with a higher risk of dementia in PD [80].

### 2.6. Visual Hallucinations

Visual hallucinations (VHs) are the most common manifestation of psychosis in PD, and have been be associated with rapid cognitive decline in PD patients. Their occurrence takes place in up to 75% of PD patients [81]. Moreover, the pathogenesis of VHs in patients with PD is not well understood. Over the course of the disease, minor hallucinations may first evolve into VHs with retained insight and, subsequently, into multimodality hallucinations with loss of insight and delusions. These usually consist of vividly perceived scenes, including people and animals. Passage hallucinations with objects passing across the peripheral visual field, extracampine hallucinations, and a sense of presence have also been described [81].

VHs in PD have been explained as a result of dysfunction of attentional networks in combination with ambiguous visual input, which may lead to VHs when remembered images intrude into consciousness [82]. Abnormal levels of the default mode network (DMN), a large-scale network that activates during rest, and in daydreaming and musing are observed in PD patients with VHs [83]. According to Weil and Reeves [81], VHs are due to over interpretation of visual input. These authors describe a reduction of white matter in posterior thalamic projections, which may play an important role for network shifting and releasing DMN inhibition [84]. 

Regarding the contribution of neurotransmitter systems in VHs, it has proved difficult to disentangle, due to the overlapping functional networks involved. It is known that levodopa treatment and dopamine agonists produce VHs [85,86]. It has also been proposed that hypersensitization of nigrostriatal dopaminergic neurons by anti-Parkinson’s drugs contribute to VHs [87]. The early disturbance of serotonergic and cholinergic pathways occurring in PD and glutamatergic and GABAergic changes affecting the overall balance between excitatory and inhibitory signaling may also play a role in the decoupling of the DMN [88]. It results in the perception of priors stored in the unconscious memory and the uncontrolled emergence of intrinsic narratives produced by the DMN [88].

VHs predict a range of poor outcomes, including more rapid cognitive decline and the development of dementia [89,90] and an increased likelihood of a move from independent living to a care home [91].

## 3. Visual Cognition Impairments in Parkinson’s Disease

Visual cognition deficits have been commonly reported in PD, although there is no consensus regarding frequency, characteristics, and relationships with other variables. Nevertheless, although many authors agree that visual cognition is not the most affected domain in PD [92,93], the majority of studies report a significant decline in visuospatial, visuoperceptive, visuoconstructive, and visual memory functions [94,95,96].

Some authors state that visual cognition deficits in PD are the consequence of central processing dysfunction rather than specific visuospatial impairments, particularly low-level perceptual deficits and executive function impairment [97,98]. Low-level visual dysfunction has important implications for understanding cognitive deterioration, as visual input is required for most of the standard neuropsychological tests. But, visual scenery generation and perception are simultaneously coupled with cognitive processes [99]. Thus, it has been reported that PD patients’ performance in a wide range of neuropsychological tests involving visual cognition can be attributed to abnormalities in low-level visual functions, especially low- and high-contrast visual acuity [96]. And, it has been suggested that lower-level vision acts as a confounder in object identification or in the time needed to interpret visual sceneries [96].

### 3.1. Visuospatial Impairment

Visuospatial deficits in PD patients have commonly been assessed with the Judgement of Line Orientation test (JLO), a tool that evaluates the ability to estimate angular relationships between line segments. Several studies have reported significant decreases in JLO scores [96,100,101,102], particularly in cognitively impaired PD patients [103]. PD patients are prone to confound oblique lines by two or more spacings [104], and show more severe intraquadrant and horizontal lines errors [105]. Some authors have reported that the interference of the visuospatial sketchpad (a component of working memory involved in the storage and manipulation of visual and spatial information) is relevant only in moderate to severe phases of the disease [106]. Recently, Kawashima et al. [107] showed that visuospatial recognition was impaired in the visuospatial o-back test, which does not involve a memory component. And, Kawabata et al. [8] have showed deficits in position discrimination in the Visual Object and Space Perception Battery (VOSP).

Mental rotation and three-dimensional and visual transformation processes have also been reported to be impaired in PD [15,108]. However, there is no consensus about mental rotation abilities in PD. Thus, some authors have documented impaired mental rotation and suggest a problem of the perception of extra-personal space [15], whereas other studies have reported spared mental rotation abilities in PD [109]. It can be argued that each mode of mental transformation is associated with a distinct network of brain regions, and these networks are likely affected differentially by the neuropathology of PD. Amick et al. [110] reported that PD patients showed an impaired ability to mentally rotate hands, but not objects. According to these authors, frontostriatal motor systems and the parietal lobes would play a necessary role for integrating visuospatial cognition with motor imagery during the mental rotation of hands. And, recently, Bek et al. [111] have proposed that PD patients would present difficulties integrating visual and kinesthetic elements of motor imagery.

### 3.2. Visuoperceptive Impairment

The Facial Recognition Test (FRT) has been used to assess the ability to recognize faces in PD patients without involving a memory component. Some PD patients, even cognitively unimpaired ones, present more difficulties on this test than on the JLO [103,112,113]. Another test, the Visual Form Discrimination Test (VFDT), has been used to evaluate visual recognition impairment in PD. Raskin et al. [114] showed a gradual impairment of visuospatial functions, and other authors have demonstrated that non-demented PD patients fail in this test [101,112]. Some authors have shown that PD patients have difficulties identifying objects embedded in complex figures and are less accurate and make more mistakes in perceptual judgements on a bistable percept paradigm (BPP) [115]. PD patients also show problems in semantical categorization of visual stimuli [116].

Kawabata et al. [8] investigated the features of visuoperceptual disturbances in PD using the battery VOSP. The authors found that one-third of patients exhibited impaired identification of incomplete letters and showed a reduction of functional connectivity in the primary visual network.

Difficulties in the perception of space and depth have also been observed in PD patients. Stereopsis impairment has been observed in some studies [117,118,119]. It has been explained as a result of basic visual perception alterations, such as color vision and contrast sensitivity deficits [118], and oculomotor behavior [120], which appear linked to the degree of disease deterioration and motor impairment [119].

Difficulties in the detection of motion are also observable in PD [121,122]. This deficit is independent of gait dysfunction and low-level vision changes, and may arise from difficulty perceptually integrating form and motion cues in posterior superior temporal sulcus [121]. These authors reported that PD patients perform significantly worse for human motion than the object motion task.

### 3.3. Visuoconstructive Impairment

Visuoconstructive impairment in the block design subtest from the Wechsler Adult Intelligence Scale (WAIS) has been related to worsening of other cognitive domains, and motor and severity in PD [123]. In the Clock Drawing Test (CDT), drawing and copy scores are significantly lower in PD, with the last correlated with high-contrast visual acuity measures [96]. Visuoconstructive abilities have also been assessed using more complex copy tests, such as the Rey–Osterrieth Copy Figure (ROCF) [123,124]. Patients show impaired visual cognition, particularly judgement of line orientation and rotation [124].

PD patients, with or without dementia, show a tendency to copy figures very close to the model, a phenomenon called “closing-in” [125]. Initially, it has been explained as a form of constructional apraxia, and some authors have proposed patients have difficulty in the visuospatial analysis of the model and/or in holding this representation in visual working memory [126]. Others suggest that the closing-in phenomenon would be an extreme manifestation of a default tendency of the motor system, so that the actions would be performed toward the focus of attention [127]. De Lucia et al. [128] have proposed that the closing-in phenomenon is related to frontal-executive impairments in PD dementia.

## 4. Side-of-Onset and Type of Parkinson’s Disease in Relation to Visual Symptoms

The side of motor symptom onset is an important consideration in the study of PD, as most patients initially present with symptoms on one side of the body, reflecting the loss of dopamine primarily in the contra-lateral hemisphere. The right hemisphere is more responsible than the left for many spatial abilities, and failure to distinguish patients with LPD from RPD may mean that visuospatial deficits that contribute to functional decline are missed in patients with LPD [129].

A factor that has been shown to influence visual processing in people with PD is the body hemifield where the first motor symptoms appeared [130] and their characteristics [131]. Thus, Verreyt et al. [130] reported that LPD patients more often perform worse on tasks of spatial attention and visuospatial orienting. Davidsdottir et al. [132] examined spatial navigation and visuospatial functioning. LPD patients were generally more visually dependent than RPD patients, who in turn were more visually dependent than the control group. Moreover, egocentric midpoint estimation was dependent on visual input biases, with the deviation increasing for LPD and decreasing for RPD. Schendan et al. [133] used a hierarchical perception task in PD, distinguishing between patients whose motor symptoms started on the left side of the body (LPDs) or the right side (RPDs). These authors observed that LPDs showed an abnormal perception of global elements, whereas RPDs perceived worse the local elements that make up an object. According to Schendan et al. [133], the link between the link side of motor symptoms and visuospatial abilities would rely on the contralateral temporoparietal junction.

On the other side, visual deficits have also been analyzed according to the type of motor symptoms that characterize the onset of the disease, defining two phenotypes: tremor dominant-phenotype (T-D) vs. bradykinesia and rigidity dominant-phenotype (B/R-D). The Visual Activities Questionnaire showed that only the B/R-D group scored significantly worse than controls in light/dark adaptation, visual acuity, depth perception, peripheral vision, and visual processing speed, whereas B/R-D only scored worse in depth perception and light/dark adaptation compared to T-D, suggesting the influence of the type of initial symptoms on visuospatial processing [134]. Other authors have noticed an increased risk of developing VHs in rigid-akinetic patients [135], whereas patients with postural instability and gait difficulty performed worse than those with T-D on visuospatial measures [131].

## 5. Gender Influence in Visuospatial Symptoms in Parkinson’s Disease

Several studies have focused on the analysis of gender differences in the manifestation of cognitive damage in patients with PD and the affected sub-processes. Gender differences have been observed on the Road Map Test of Direction Sense, a right–left discrimination task that requires egocentric mental rotation in space, with men being superior [136]. Davidsdottir et al. [132] examined spatial navigation and visuospatial functioning. Gender differences were found in the navigation task, egocentric midline test, line bisection, and motion perception. Oltra et al. [137] observed that female patients had lower scores than males in the JLO. In the same line, Pasotti et al. [138] analyzed 306 patients of both sexes, observing gender differences in the execution of cognitive tasks, with males superior in visuospatial tasks. These differences were more noticeable in the initial stages of the disease, with the presence of estrogens in dopaminergic neurons being a possible protective factor against cognitive deterioration in this disease. In this line, Crucian et al. [108] reported that men with PD demonstrated significantly lower scores on the Mental Rotations Test than men of similar age and education, whereas PD and control women performed at a similar low level.

Nevertheless, recent research has reported that there was no interaction between sex and Parkinson’s diagnosis (with or without mild cognitive impairment) for visuospatial function [139]. Other studies have reported no male–female differences, including Cronin-Golomb and Braun [140] on visuospatial problem solving using Raven’s Coloured Progressive Matrices; Amick et al. [110] on mental rotation; and Schendan et al. [133] on hierarchical pattern perception, a test of global and local visual pattern processing. The latter two studies reported LPD-RPD effects. Thus, several studies suggest that gender differences pertain to some, but not all, visuospatial abilities, and may interact with the side of disease onset [129].

## 6. Neuroanatomical Correlates of Visuospatial and Visuoperceptive Deficits in Parkinson’s Disease

Visuospatial (VS) and visuoperceptive (VP) deficits in PD have been related to cortical thinning in the parieto-occipital and fronto-temporal networks, along with structural disturbances in antero-posterior white matter pathways (Table 1). Moreover, as brain degeneration progresses, there is a worsening in VS/VP performance, reaching its worst level with the onset of cognitive impairment [141,142] (Table 2).

Several structural magnetic resonance image (sMRI) studies have found a correlation between neuropsychological test scores and cortical thinning in PD. Pereira et al. [112] found that the gray matter density in the superior parietal and superior occipital gyrus correlated with visuospatial performance in PD, whereas reduced gray matter in the fusiform, the parahippocampus, and the middle occipital gyrus was associated with poor performance on visuoperceptual tests. These authors described the relationship between facial recognition deficits using the FRT and gray matter thinning in the fusiform gyrus (BA 19, 36), and clusters in the parahippocampal region, the middle occipital gyrus (BA 19), and the inferior frontal gyrus (BA 47). Using the same task, Garcia-Diaz et al. [141] described correlated patients’ performance with cortical thinning in the left lateral occipital area. On the other side, using the visual form discrimination test (VFDT), Pereira et al. [112] observed that performance correlated with gray matter reductions in the bilateral superior parietal lobes (BA 7, 40) and the superior occipital (BA 19), the middle frontal (BA 9), and the inferior frontal gyrus (BA 47). These results would indicate different patterns in gray matter reduction in visual associative areas for facial recognition and visual form discrimination. Facial recognition would be related to gray matter reductions in areas of the ventral occipitotemporal cortex, whereas visual form discrimination would in volve dorsal parietal areas. According to the authors, facial recognition impairmentwould correlate with the medial temporal lobe, and impaired visual form discrimination with superior parietal regions. Occipitotemporal and occipitoparietal pathways send projections to the prefrontal cortex, where spatial information is maintained “online”.

Filoteo et al. [143] have reported a decreased volume bilaterally in the superior temporal cortex and the right lateral occipital cortex (both in the object-based system), with poorer performance in the JLO.

Poorer visuoconstructive performance on the Pentagon Copy test (PCT) has been correlated with decreased volume in the frontal regions (right Supplementary Motor Area, left rostral middle frontal cortex, and pars triangularis), as well as in the object-based system (left Cuneus) [143].

Cognitively impaired PD patients show a more pronounced and extended pattern of cortical atrophy. Rektorova et al. [144] observed gray matter volume reductions in the hippocampus, amygdala, and neocortical temporal regions related to impairment in visuospatial abilities in demented PD patients. More recently, Garcia-Diaz et al. [141] found that MCI-PD patients exhibit significantly greater progressive cortical thinning in left lateral occipital and inferior parietal regions, and in right medial temporal regions. The authors reported that scores on the PCT correlated with a cluster in the left entorhinal region that included the middle and inferior temporal gyri, the medial temporal pole, and the parahippocampal, fusiform, lingual, and lateral occipital cortices. Their performance on the JLO was related to cortical atrophy in clusters in the left insula, inferior, and superior temporal areas, and the right fusiform gyrus, which extended to the left temporal pole, entorhinal, fusiform, and lingual cortices. FRT scores correlated with cortical thinning in the left lingual gyrus. Similarly, the Symbol Digit Modalities Test (SDMT) correlated with a diminution in the left superior temporal, parahippocampal, and lingual, and the right parahippocampal cortices. According to the authors, this anatomical pattern of cortical atrophy was valid when only patients with sustained cognitive impairment at the 4-year follow-up were included. Lee et al. [145] have also described different patterns between amnestic MCI-PD patients (the most common MCI subtype) and non-amnestic MCI-PD patients. The authors reported that visuospatial impairment was more severe in the amnestic group, and a direct comparison between both groups showed a decreased gray matter density in the bilateral precuneus, left primary motor, and right parietal areas.

The side-of-onset also influences the gray matter density pattern in relation to visuospatial alterations. Thus, left PD patients have greater visuospatial impairments compared to right PD patients and lower gray matter volume in the right Dorsolateral Prefrontal Cortex, regardless of other variables, such as age or premorbid cognitive status [146].

Another marker of cortical degeneration in PD related to VS/VP impairment is brain asymmetry. Segura et al. [101] described an initial deterioration of right temporo-parietal regions, followed by a progressive bilateral hemisphere degeneration in PD. As mentioned above, lateralized motor symptoms onset has been studied as a variable that modulates visuospatial skills deterioration.

White matter loss also contributes to worsening visuospatial performance in PD [147]. In the study by García-Díaz et al. [7] on a sample of PD patients, with and without cognitive impairment, the authors reported correlations between VS/VP scores and white matter fractional anisotropy values in the corpus callosum, bilateral forceps minor, uncinate fasciculus, inferior frontooccipital fasciculus, forceps major, and inferior longitudinal fasciculus. All the VS/VP tests studied showed significant correlations between their scored and fractional anisometropia values, but it was larger for the SDMT.

## 7. Functional Neuroimage Correlates of VS/VP Deficits in Parkinsons’ Disease

Most functional neuroimaging studies (functional MRI, positron emission tomography PET, and single-photon emission computerized tomography SPECT) have reported altered activation, altered blood flow, or reduced metabolism in both dorsal and ventral visual pathways, which probably indicates an alteration in the normal bottom–top visual processing and the presence of aberrant top–down visual processing [148].

Early studies based on PET imaging by Eberling et al. [149] and Bohnen et al. [150] showed a clear reduction of cerebral glucose metabolism in visual association, primary visual, and right parietal cortices in non-cognitively affected patients. Furthermore, lower performance in visuospatial tasks has been associated with fluoro-deoxyglucose hypometabolism or hypoperfusion in occipital and frontal cortices of non-cognitively impaired subjects [151], along with lower levels of 123I-iodoamphetamine single-photon emission computed tomography-based assessment in right hemisphere posterior-frontal cortices [152].

With disease progression, incident dementia in idiopathic PD is announced by metabolic changes within visual association (BA 18) and posterior cingulate cortices. Findings indicate that incident dementia preferentially involves BA18, whereas reductions in the primary visual cortex (BA 17) can be seen in PD without dementia. The Benton Visual Retention test, which assesses visuospatial perception, visuomotor and visuoconstructive abilities, and visual memory, best correlates with BA 18 metabolism [153]. Extension of occipital hypometabolism from the primary to the visual association cortices, together with precuneus hypometabolism, may be the early cortical metabolic “signature” of incident dementia in PD, with visual association cortex hypometabolism linked to the presence of VHs in more advanced PD [153].

Using functional magnetic resonance (fMRI), Caproni et al. [154] found that PD patients had reduced activation of the right insula, left putamen, bilateral caudate (in particular, in the right hemisphere), and right hippocampus, together with greater activation of the right dorso-lateral prefrontal cortex (DLPFC) and bilateral posterior parietal cortex (PPC) during visuospatial judgement. The authors propose that the DLPFC activation reflex is a compensatory mechanism, through continuous control by the top–down visual processing system.

Alteration in PD brain function related to VS/VP deficits has also been observed via non-invasive resting state fMRI, supporting the idea of a marked fronto-occipital-parietal dysfunction in the right hemisphere [155], which is more noticeable as the disease progresses [156], and is responsible for VS/VP impairments [157]. Although such reduction of right-hemisphere functional connectivity is observable in early stages, it progresses and affects bilateral prefrontal and frontoparietal networks when mild cognitive impairment (MCI) and/or dementia appear [158], accompanied by increased Regional Homogeneity (ReHo) in the medial-superior occipital gyrus compared to healthy controls [159].

## 8. Neuroanatomical Correlates of Parkinson’s Disease Visual Hallucinations

Imaging studies of VHs in PD to date have been based on relatively small samples and have used different designs to control for the degree of cognitive decline, stage of PD, and dopamine medication, showing little consistency across studies [160].

A number of structural neuroimaging studies have analyzed the cerebral basis of VHs in PD. Gray matter atrophy has been described in multiple regions of the brain, such as the primary visual and visual association cortices; limbic regions, and cholinergic structures, such as the pedunculopontine nucleus and substantia innominata, which are involved in visuospatial-perception, attention control, and memory [148].

Widespread reductions in the cortical thickness of PD patients with hallucinations have been identified in the occipital, parietal, temporal, frontal, and limbic lobes of PD patients with VHs. However, not all regions are equally affected, and, notably, there appears to be a posterior asymmetry, with relative sparing of the left ventral visual stream (ventral occipitotemporal cortex) compared to the homologous region in the right hemisphere [161]. Of these, the cuneus and superior frontal gyrus bilaterally emerged as the dominant components.

Ramírez-Ruiz et al. [162], in a VBM study, reported reduced GM volume in the left lingual gyrus and bilateral superior parietal lobule in PD patients with VHs compared to those without VHs. Pagonabarraga et al. [163] have proposed a progressive volume reduction from the unilateral left superior parietal in minor hallucinations (mHs) to the bilateral superior parietal in VHs. A significant reduction of GM volume has been observed in the right inferior frontal, left temporal, and thalamic areas [164]; the left opercula frontal gyrus and left superior frontal gyrus [165]; the cingulate [166]; and in the bilateral dorsolateral prefrontal cortex, rostral part of prefrontal cortex, bilateral primary visual cortex, and regions corresponding to the secondary visual cortex, such as the left inferior occipital gyrus, right lingual cortex, right supramarginal gyrus, and left fusiform gyrus [167]. Vignando et al. [161] report that regions with reduced thickness for higher severity scores (negative correlation) were found in the posterior parietal, posterior cingulate, and superior temporal cortex. It has also been observed a degenerative process in the head of the hippocampus in hallucinating PD patients [168] that would involve the whole hippocampus and cause dementia in hallucinating PD patients. The atrophy of cholinergic structures, such as the pedunculopontine nucleus (PPN) and its thalamic projections, has also been associated with VHs in PD [169], the substantia innominata (SI) [164]. Finally, reduced volume in cerebellar lobules VIII, IX/VII, and Crus 1 has been associated with VHs in PD [170].

Goldman et al. [166] observed that gray matter atrophy occurred both in the ventral (what) and dorsal (where) pathways, responsible for object and facial recognition and identification of the spatial locations of objects. Thus, those patients who experienced VHs exhibited gray matter atrophy in the cuneus, lingual, and fusiform gyri; middle occipital lobe; inferior parietal lobe; and cingulate, paracentral, and precentral gyri. These structural changes were not related to the presence of dementia.

## 9. Functional Neuroimage Correlates of Parkinsons’ Disease Visual Hallucinations

A seminal fMRI study by Stebbins et al. [171] reported that VHs in PD patients reflect an abnormally increased activation in anterior cortical regions, such as the inferior frontal cortex and caudate nucleus, accompanied with reduced activation in the parietal lobe and cingulate gyrus, which has been explained as a shifting of attentional visual circuitry from posterior (down–top) to anterior (top–down) regions. More recently, Yao et al. [83] reported an increased functional connectivity in the default mode network (DMN) in the right middle frontal gyrus and bilateral posterior cingulate gyrus/precuneus of VH PD patients in comparison to non-VH patients. These data support the hypothesis of an excessive and aberrant top–down processing.

PET studies have reported an increased glucose metabolism in frontal regions (the left superior frontal gyrus) in hallucinating PD patients [172], and a decreased metabolism in the occipito-parieto-temporal region (sparing the occipital pole) that included both dorsal and ventral visual streams [173]. Park et al. [174] has reported a reduced metabolism in both visual streams of hallucinating PD patients, but predominantly in the ventral one. In the same line, a SPECT study by Oishi et al. [175] reported increased perfusion in the right superior and middle temporal gyri in hallucinating PD patients. The increased perfusion of the superior temporal gyrus is in line with the hypothesis of prevalent top–down visual processing. Also, the fronto–striatal circuit involved in the dorsal attention network has been involved in the pathogenesis of VHs in PD [176]. Kiferle et al. [177] have suggested that frontal impairment observed in PD patients with VHs may be due to a right caudate dysfunction, reflecting an impairment of cortico–subcortical circuits.

**Figure 2:**
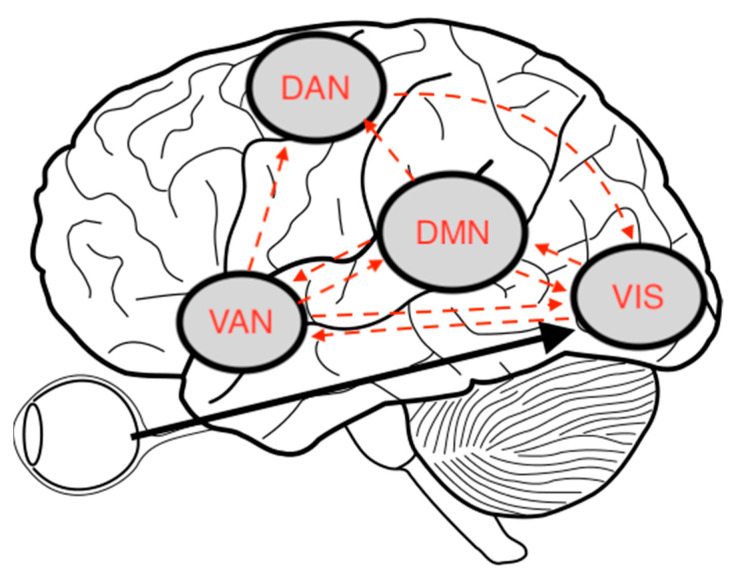
The van Ommen [182] model of visual hallucinations. Complex VHs are the result of a dissociation of higher-order visual processing areas from the primary visual cortex. Simultaneously, a looping of brain activity across the outside-world-focused DAN, the inner-world-focused and memory-related DMN, and the saliency-focused VAN, bias conscious visual perception away from information coming from the outer world and towards internally generated percepts. Visual network (VIS). Modified from “Human Brain sketch with eyes and cerebrellum.svg”, work released into the public domain by Hankem. https://commons.wikimedia.org/wiki/File:Human_Brain_sketch_with_eyes_and_cerebrellum.svg (accessed on 11 July 2023).

Dysregulation of the ventral attentional network (VAN), dorsal attentional network (DAN), and default mode network (DMN) have been implicated in models of VHs in PD [178] with reduced activity in the DAN [82]. PD patients with hallucinations show widespread disruption in structural connections, which particularly affect highly connected brain regions or “hubs” required for brain transitions between different cognitive states [179,180] (See Figure 2). Zarkali et al. [181] found that PD patients with hallucinations show impaired temporal dynamics, with a predisposition towards a predominantly segregated state of functional connectivity, and require less energy to transition from the integrated to the segregated state. Moreover, the thalamus and regions within the DMN are critically involved in the network imbalance in PD hallucinations.

## 10. Neurochemistry of VS/VP Deficits in Parkinsons’ Disease

Dopaminergic neurons are diffusely present within the retina. Dopamine is present in the retina in amacrine cells of the inner border of the inner nuclear layer and in interplexiform cells, influencing the activity of photoreceptors, ganglion cells, and bipolar cell receptive fields [183,184]. The role of dopaminergic reduction in the basal ganglia and the impact on the arterial walls of the frontal cortex would explain the appearance of problems in eye movements and in pupil reactivity in PD [27,185]. Reductions in dopamine levels in the basal ganglia and frontal cortex may also deplete levels in the superior colliculus and, therefore, could be a factor in the production of defective saccades [186]. Furthermore, it has been observed that dopaminergic medication adversely impacts visuoperceptual performance, with it worse in the *on* compared to the *off* medication state. Thus, dopaminergic medication can ameliorate movement deficits, but reduces visuoperceptual accuracy because of overdosing [187]. Finally, it must be also noted that although all dopaminergic drugs have been associated with incident VHs, this association has been shown contradictory [163], and other factors could be considered responsible [188].

The imbalance of the pedunculopontine cholinergic projections on the visual cortex produces aberrant visuospatial processing [123]. Moreover, the appearance of abnormal VP phenomena, such as hallucinations in people with PD, has been associated with degeneration of the nucleus basalis of Meynert and its cholinergic projections between the brainstem and the frontal cortex, also affecting the execution of visuospatial tasks [189,190].

In addition to the dopaminergic–cholinergic balance, VHs have been associated with decreased occipital GABA in PD [191]. Whether this system is affected in the retina and visual cortex of all PD patients, and from early stages, remains to be determined.

Regarding other neurotransmission systems, the serotonergic system also seems to play a key role in the manifestation of VHs and visuoperceptual function in PD. Thus, VHs and other visual-perceptual dysfunctions have been observed in PD associated with a decrease of the serotonergic receptor 5-HT2A in the right insula, bilateral dorsolateral prefrontal cortex, right orbitofrontal cortex, right middle temporal gyrus, and right fusiform gyrus (Cho et al., 2017). Vignando et al. [161] reported an association for 5-HT2A and 5-HT1A confined to regions linked to VHs, rather than the cortex as a whole, suggesting the neurotransmitter effects were specific to VHs, suggesting the possibility that degeneration in these neurotransmitter systems in PD underlies synaptic loss and cortical thinning. Cho et al. [192] have also reported that 5-HT2A correlated with visuoperceptual function. In particular, the FRT score was related to receptor binding in the right anterior cingulate cortex (ACC), left DLPFC, and inferior temporal gyrus. A reduced binding in ACC and other prefrontal regions was related to a lower VOSP total score. The author also described negative correlations between 5-HT2A in the middle/inferior temporal gyrus and Rey Complex Figure copy scores, as well as between the occipital BA 18 and JLO scores. These regions are part of the ventral visual system within the bottom–up network.

## 11. Genetic Factors of VS/VP Deficits in Parkinsons’ Disease

Although genetic factors are only implicated in a minority of cases of PD, genetic approaches could contribute to understanding the etiology and manifestation of VS/VP impairments in PD. Mutations in the leucine-rich repeat kinase 2 (LRRK2) and parkin RBR E3 ubiquitin protein ligase genes are thought to be protective against cognitive impairment and ocular disturbances [193]. Moreover, lysosomal enzyme glucocerebrosidase (GBA) mutation carriers show poor visuospatial performance [194,195] and a higher frequency of hallucinations [196]. Moreover, it has been reported that microtubule-associated protein tau (MAPT) H1 haplotype carriers present a higher risk of dementia, are less accurate with difficult spatial rotations, and show lower activity in the parietal cortex and caudate nuclei [197].

## 12. Conclusions

Numerous visual and perceptual problems have been observed in patients with PD. However, these problems are usually under-recognized and poorly understood, leading to a lack of appropriate treatment.

Different structures and networks have been involved in visual deficits in PD, from lower-level structures, such as the retina, to visual pathways involved in higher-level visual cognition. Thus, low-level visual dysfunction, like visual acuity and contrast sensitivity impairment, has been extensively observed in PD. And, as most of the standard neuropsychological measures rely on visual input for assessing cognitive functions, it is observed that lower mechanisms’ deficits also affect higher cognitive capabilities. Moreover, visual scenery generation and perception are also limited by cognitive processes that are deteriorated in PD, such as attention [99]. As result, the incoming visual information is constantly regulated and tuned by top–down processes [154].

One of the main approaches to study the causes of VS/VP deficits in PD has been the structural neuroimage. Studies have shown alterations in the cortical thickness of bilateral temporo–parietal–occipital areas and widespread posterior–anterior white matter microstructure alterations [7,198]. In addition, bilateral degeneration of posterior cortical regions is associated with a progressive worsening in VS/VP performance. And, it is also observed a progressive cortical volume reduction in posterior parieto-temporal regions of PD-MCI in comparison with cognitively unimpaired PD subjects [141].

Functional alterations in both cortical areas and subcortical regions involved in VS/VP have been also observed in PD. In particular, a reduced activation in the right insula, left putamen, bilateral caudate, and right hippocampus, as well as an over-activation of the right dorso-lateral prefrontal and the posterior parietal cortices, particularly in the right hemisphere, have been observed in PD. Moreover, a loss of cortical efficiency and compensatory mechanisms during visual processing have also been observed in PD patients [143,154]. It has been observed that PD patients showed greater activations of right DLPFC and bilateral PPC. Both of these regions, together with frontal–striatal circuits, are known to be part of the “Top–Down” visual processing system, which is involved in the selection and organization of complex visual information. DLPFC greater activation has been considered a compensatory response in PD patients, involving a continuous control by “Top–Down” mechanisms associated with visual working memory. Moreover, the greater activation of bilateral PPC could be necessary to overcome the initial impairment of the network [154].

Some authors have also suggested a role for lateralization of the basal ganglia circuits in stimuli perception, associated with a different clinical manifestation in left- and right-side PD onset [133]. Thus, right-sided onset PD is characterized by impairment in local-level processing, a consequence of left frontal and parietal deficits, whereas left-sided onset PD is characterized by an alteration of global-level processing, due to right parietal dysfunction [110,133].

Research about neurochemical alterations associated with VS/VP disturbances in PD is scarce, and many times the results have been contradictory. Different studies have reported the dopaminergic basis of retinal and other neurodegenerative pathologies in PD related to visual and ocular alterations [27,185], whereas visuoperceptive and visuospatial impairments correlate with abnormalities in several interrelated neurotransmitter systems, mainly the dopaminergic, noradrenergic, serotonergic, and cholinergic systems [189,199].

Visuoperceptual and visuospatial deficits in PD correlate to disease progression and have been proposed as an early marker of cognitive deterioration [132]. For instance, cross-sectional correlational studies have established a relationship between the degree of atrophy in posterior brain regions and VS/VP impairment [141]. Moreover, visuoperceptual deficits have also been associated with VHs, which are also considered predictive of disease progression.

VHs are part of the syndrome of PD psychosis, and one of the most debilitating symptoms for patients’ quality of life. Although, the precise pathophysiology of VHs in PD remains unclear, neuropathological and structural imaging studies have provided some insight [199]. Thus, neuroimaging studies have revealed gray matter atrophy in multiple regions, with the most prominent changes across areas involved in visual perception (including the ventral ‘what’ and dorsal ‘where’ pathways), the hippocampus, and several cholinergic brain structures, such as the substantia innominata and pedunculopontine nucleus [164,166]. It has been considered that dentification of brain structures associated with VHs in PD may permit earlier detection of at-risk patients and ultimately development of new therapies for targeting hallucinations and visuoperceptive functions [166].

As detailed above, the etiology and neurobiological bases of visual and perceptual impairments in PD are complex and multifaceted, and the same could be said with regard to their association with motor symptoms. Ocular, visual, visuoperceptive, and visuospatial deficits can occur independently in PD and may depend on multiple factors. Thus, each individual with PD may present a unique combination of these impairments, with variable symptomatology. Moreover, these alterations are hypothesized to play a role in the etiology of the main motor signs of PD, such as the freezing of gait [200]. Therefore, to reveal the particular mechanisms behind visual symptomatology and the potential therapeutic strategies poses a tremendous challenge for future research. The combination of genetics and functional neuroimaging can provide a promising strategy for classification and identification of potential biomarkers, which can be tested in future clinical trials designed to fight and prevent PD.

## Figures and Tables

**Table 1:** Neuroanatomical substrate of visuoperceptual, visuospatial, and visuoconstructive impairment in PD.

Domain	Task	Cortical Region
Visuoperceptual	Facial Recognition Test (FRT) [112,141] Visual Form Discrimination Test (VFDT) [112]	Fusiform Gyrus (BA 19, 36) Parahippocampal Middle Occipital Gyrus Inferior Frontal Gyrus (BA 47) Left Lateral Occipital Bilateral Superior Parietal (BA 7, 40) Superior Occipital (BA 19) Inferior Frontal Gyrus (BA 47)
Visuospatial	Judgement Line Orientation Task (JLO) [143]	Bilateral Superior Temporal Right Lateral Occipital
Visuoconstructive	Pentagon Copy Test (PCT) [143]	Right Supplementary Motor Left Rostral Middle Frontal Pars Triangularis Left Cuneus

**Table 2:** Neuroanatomical substrate of cortical thinning as a possible biomarker of evolution to dementia [141].

Domain	Task	Cortical Region
Visuoperceptual	Facial Recognition Test (FRT) Symbol Digit Modalities Test (SDMT)	Left Lingual Gyrus Left Superior Temporal Left Parahippocampal Left Lingual Gyrus Right Parahippocampal
Visuospatial	Judgement Line Orientation Task (JLO)	Left Insula Inferior Temporal Superior Temporal Right Fusiform Gyrus
Visuoconstruction	Pentagon Copy Test (PCT)	Left Entorhinal Middle and Inferior Temporal Gyri Medial Temporal Pole Parahippocampal Fusiform Cortex Lingual Cortex Lateral Occipital Cortex

## Data Availability

No new data were created.
